# Current Trends in the Molecular Classification of Renal Neoplasms

**DOI:** 10.1100/tsw.2006.390

**Published:** 2006-12-15

**Authors:** Andrew N. Young, Viraj A. Master, Mahul B. Amin

**Affiliations:** ^1^ Department of Pathology and Laboratory Medicine, Emory University School of Medicine, Atlanta, GA, USA; ^2^ Department of Urology, Emory University School of Medicine, Atlanta, GA, USA; ^3^ Department of Pathology and Laboratory Medicine, Cedars-Sinai Medical Center, Los Angeles, CA USA

**Keywords:** kidney neoplasms, gene expression profiling, microarrays

## Abstract

Renal cell carcinoma (RCC) is the most common form of kidney cancer in adults. RCC is a significant challenge for pathologic diagnosis and clinical management. The primary approach to diagnosis is by light microscopy, using the World Health Organization (WHO) classification system, which defines histopathologic tumor subtypes with distinct clinical behavior and underlying genetic mutations. However, light microscopic diagnosis of RCC subtypes is often difficult due to variable histology. In addition, the clinical behavior of RCC is highly variable and therapeutic response rates are poor. Few clinical assays are available to predict outcome in RCC or correlate behavior with histology. Therefore, novel RCC classification systems based on gene expression should be useful for diagnosis, prognosis, and treatment. Recent microarray studies have shown that renal tumors are characterized by distinct gene expression profiles, which can be used to discover novel diagnostic and prognostic biomarkers. Here, we review clinical features of kidney cancer, the WHO classification system, and the growing role of molecular classification for diagnosis, prognosis, and therapy of this disease.

## CLINICAL FEATURES OF KIDNEY CANCER

In 2006, there will be 38,890 newly diagnosed cases of renal cell carcinoma (RCC) with 12,840 deaths, according to the American Cancer Society. RCC accounts for 3% of all human malignancies[1]. The incidence of RCC is greatest in developed societies[2], perhaps due to highly prevalent risk factors, such as cigarette smoking, obesity, hypertension, and analgesic use. Lipid peroxidation has been proposed as a unifying etiologic mechanism for these risk factors[3,4,5,6]. Today, due to frequent use of abdominal imaging, many cases are discovered incidentally at organ-confined stage[7]. Open total nephrectomy is standard therapy for localized RCC, but laparoscopic surgery, with nephron-sparing tumor resections or ablations, are used increasingly for small (<4 cm) lesions, as well as other clinical situations[8,9,10,11]. Although surgery is potentially curative for organ-confined RCC, one-third of such lesions metastasize after therapy. Furthermore, despite trends toward earlier diagnosis, 40% of cases present with extrarenal growth or metastases[12].

RCC is highly variable in terms of clinical behavior. Pathologic stage using the tumor-node-metastasis (TNM) system is a critical prognostic factor[2], and recent refinements to the TNM classification have been proposed to optimize correlation between outcome and tumor size and local extension[13,14]. In an attempt to further improve prediction, several groups have developed prognostic models for metastatic or postnephrectomy RCC. These models combine pathological findings with clinical parameters, such as performance status and laboratory values; important examples include the UCLA integrated staging system (UISS), the Memorial Sloan Kettering nomogram, and the Mayo Clinic Stage, Size, Grade and Necrosis Score (SSIGN)[15,16,17,18,19,20]. In addition to these clinical models, elevations in immune markers, such as erythrocyte sedimentation rate and C-reactive protein, have been shown to carry negative prognostic significance[21,22,23]. Recently, our collaborators discovered that perioperative thrombocytosis is a negative prognostic factor in RCC[24,25], an observation now confirmed by several independent investigators[26,27,28].

Metastatic disease from RCC typically manifests in the lung, bone, brain, abdominal viscera, the contralateral kidney, adrenal glands, and regional lymph nodes[29]. However, RCC may metastasize to unusual locations and present as metastatic carcinoma of unknown primary[2]. Most modern series cite the lung as the most common site of metastases (~1/3), followed by bone (~20%), liver (~20%), and brain (~10%), and overall survival may correlate with site of metastasis. Currently, a need exists for biomarkers to predict a biological propensity for metastasis and likely sites of spread, in order to provide patients with accurate prognosis, tailor surveillance to detect early relapse in patients at risk, and design targeted molecular therapy.

Resection of solitary metastases from RCC can be accomplished for protracted 5-year survival rates in the range of 35–50%. Selection criteria are poorly defined. In general, favorable predictors for long-term survival are young age, single site of first recurrence, curative resection of first metastasis, long disease-free interval from initial nephrectomy, and pulmonary recurrence. Kavolius et al. reviewed the Memorial Sloan-Kettering experience of 278 patients with recurrent RCC[30]. In this series, disease free interval of >12 months was the strongest predictor of overall survival (55% vs. 9% 5-year overall survival $p < 0.0001$), followed by solitary site of metastases (54% vs. 29%, $p < 0.001$), age <60 years (49% vs 35%, $p < 0.05$), and lung relapse. The clinical impact of RCC bone metastasis is a topic of growing interest[31,32,33,34,35,36,37]. Surgical resection is challenging due to association with significant hemorrhage and possible reconstruction. Prior to the publication of Althausen et al.[35] in 1997, the prevailing dogma was that RCC with skeletal metastasis had poor prognosis, which led to the general approach of palliative treatment of bone lesions. However, Althausen's study of 34 patients, followed for a mean duration exceeding 7 years, showed that a subset of patients could achieve disease-free status of 55% at 5 years and 39% at 10 years. Absence of metastatic disease at initial presentation, and long disease-free interval from nephrectomy to bony metastasis were correlated with better outcomes. Metastases to the axial skeleton, especially the pelvis, were significantly worse, in terms of overall survival then metastases to the appendicular skeleton (extremities). Other, modern series, such as Durr et al.[32], have continued to demonstrate these results. Fuchs et al.[37] indicated that wide surgical excision of a solitary bony metastasis from RCC is not absolutely mandatory to improve survival, but, in their series, 15% of patients treated with stabilization alone without resection had local progression. Thus, wide resection of metastatic lesions coupled with bone stabilization may be necessary to prevent local disease progression and complications. Based on these observations, biomarkers to predict likelihood of bone metastasis and response to metastatectomy could have significant clinical impact.

For widely metastatic disease, systemic anticancer agents are the standard treatment, as reviewed elsewhere[38]. RCC is often resistant to standard chemotherapy. The cytokines interleukin-2 and/or interferon-alpha are FDA approved for treating advanced RCC, but response rates are poor and 5-year survival remains less than 10%[39]. It remains difficult to predict response to systemic therapy. In light of these limitations, interest is growing in the molecular lesions that characterize kidney tumors, as an approach to define novel targets for molecular-based therapy and monitoring[40,41,42,43]. Molecular classification for diagnosis, prognosis, and treatment is the focus of a growing body of research described in subsequent sections of this review.

## PATHOLOGIC FEATURES OF KIDNEY CANCER: WHO CLASSIFICATION

The current system for renal tumor classification was published in 2004 by the World Health Organization (WHO)[2]. The WHO classification system separates tumors into histologic subtypes based on distinctive light microscopic morphology, clinical behavior, and underlying genetic mutations. Most cases fall under the headings of clear cell RCC (75% of surgically removed renal tumors), papillary RCC (10%), chromophobe RCC (5%), and renal oncocytoma (5%). Angiomyolipoma is the most common adult mesenchymal tumor (1%), while nephroblastoma (Wilms tumor) is the most common pediatric renal malignancy. The WHO system also includes newly recognized, rare tumors defined by clinical, morphologic, and genetic factors including carcinoma of the collecting ducts of Bellini, renal medullary carcinoma, renal carcinoma associated with Xp11.2 translocations, and mucinous tubular spindle cell carcinoma[2]. Carcinomas arising in end-stage renal disease, as well as thyroid-like and tubulocystic carcinomas, have been proposed as distinct clinicopathologic entities[44,45,46,47].

The gross and microscopic appearance of renal tumors are quite variable, but many cases can be diagnosed reliably based on the following characteristic criteria[2,12]. Clear cell RCC is usually an infiltrating mass with solid, alveolar or acinar growth patterns, and “clear” tumor cells associated with anastomotic blood vessels. Cystic clear cell tumors also occur. Papillary RCC is a circumscribed, encapsulated mass with neoplastic cells in papillary growth pattern, admixed with foam cells and necrosis. This subtype is further divided by some investigators into Type 1, which exhibits papillae lined by single layers of small tumor cells with scant cytoplasm, and Type 2, which contains tumor cells with higher nuclear grade, eosinophilic cytoplasm and pseudostratified nuclei on papillary cores[2]. Chromophobe RCC contains tumor cells in an alveolar or nested growth pattern, containing irregular nuclei, perinuclear halos, and prominent cell borders. In some cases, larger cells with flocculent cytoplasm are intermixed with smaller, granular eosinophilic cells. In other cases, eosinophilic cells are predominant. Oncocytoma is a circumscribed mass (often with central scar), with nested or tubulocystic growth of “oncocytes” containing round nuclei and granular eosinophilic cytoplasm. Oncocytoma can be difficult to distinguish from eosinophilic chromophobe RCC. Indeed, pathologic classification of any RCC can be difficult because all subtypes may exhibit solid, alveolar or papillary growth patterns, contain neoplastic cells with clear or granular cytoplasm, or display high-grade, sarcomatoid histology. Diagnosis is particularly challenging with small tumor biopsies from nephron-sparing procedures (see above), which provide limited histologic information and are prone to sampling error.

WHO classification is emerging as an important predictor of clinical behavior and therapy[2,12]. Among common RCC subtypes, clear cell RCC has the highest rate of extrarenal growth, metastasis, and mortality[2,12]. Cytokine therapies are most effective for this subtype, and clinicians often confirm clear cell histology before initiating treatment[48]. In addition, antiangiogenic agents are under investigation for clear cell RCC, targeting tumor vascularity[49]. Papillary and chromophobe carcinomas are indolent, but have potential to metastasize or transform to high-grade, sarcomatoid cancers. Papillary RCC has the highest rate of multifocality (in particular Type 1 variants) and association with end-stage renal disease[12,50]. Systemic therapy is not standardized for metastatic RCC with nonclear cell histology[51]. Oncocytoma is closely related to chromophobe carcinoma, but is consistently benign[12,52]. Although rare, collecting duct carcinoma is a very aggressive malignancy with the highest tendency to metastasize of all renal epithelial tumors[2]. Patterns of metastasis may differ among tumor subtypes, with papillary and chromophobe RCC relatively likely to spread to lymph node and liver, respectively[53].

## GENETICS OF KIDNEY CANCER

Renal tumor subtypes are associated with distinct, reoccurring cytogenetic abnormalities[54] and hereditary cancer syndromes[55]. Hereditary tumors often occur multifocally at earlier age, and suspected cases can be diagnosed with a range of clinical genetic assays on patient germline[56]. In contrast, sporadic RCC is tested only infrequently by cytogenetics due to technical difficulty and limited diagnostic sensitivity. Clear cell RCC is associated with von Hippel-Lindau (VHL) disease, an autosomal dominant tumor susceptibility syndrome involving the VHL tumor suppressor gene on chromosome 3p25. In addition, VHL loss of function is the most common genetic defect in sporadic clear cell RCC. VHL mutations promote tumor hypervascularity by impeding degradation of the angiogenesis regulator hypoxia-inducible factor-1 alpha (HIF1A)[57]. Clear cell RCC also arises in the rare syndrome of constitutional chromosome 3 translocations, mapped to various breakpoint sites distinct from the VHL locus[58]. Inherited papillary RCC occurs in the autosomal dominant hereditary papillary renal carcinoma syndrome (HPRC), caused by germline activation of the hepatocyte growth factor receptor gene (MET) on chromosome 7q31, and the hereditary leiomyomatosis and renal cell cancer syndrome (HLRCC), affecting the fumarate hydratase gene (FH) on chromosome 1q42.1[59]. Papillary carcinomas in HRPC tend to be Type 1 variants, while those in HLRCC tend to be Type 2 and more aggressive[2]. In sporadic papillary RCC, trisomy 7 and 17, and loss of Y, are the most common chromosomal defects[54]. Chromophobe RCC and oncocytoma arise in Birt-Hogg-Dubé syndrome, an autosomal dominant, multiorgan system tumor syndrome mapped to chromosome 17p11.2[60]. In sporadic chromophobe RCC, the most characteristic genetic lesion is loss of multiple chromosomes, while sporadic oncocytoma tends to exhibit different mutations[54]. Angiomyolipoma is associated with tuberous sclerosis, an autosomal dominant disorder caused by loss-of-function mutations in the TSC1 and TSC2 tumor suppressors on chromosomes 9q34 and 16p13, respectively[2].

## MICROARRAY TECHNOLOGY

Several experimental methods have been developed to analyze global mRNA expression in biological samples; each method has been applied to human cancers for the purpose of tumor classification. High-density expression microarrays have emerged as the predominant method. Microarrays are solid matrices containing several thousand nucleic acid hybridization targets, representing a large fraction of the entire expressed genome, at fixed addresses. Two major micorarray platforms include spotted microarrays, containing purified cDNAs or oligonucleotides printed robotically onto glass slides[61,62], and microarrays with short oligonucleotides synthesized directly onto solid substrates using photolithographic or inkjet techniques. The photolithographic approach was pioneered by Affymetrix[61,63]. Alternate expression profiling methods include differential display and serial analysis of gene expression. Technical aspects of microarrays and alternate expression profiling systems are described elsewhere[64,65,66].

In most microarray studies of cancer, the number of mRNA species analyzed exceeds the number of tumor samples by several orders of magnitude. Therefore, data tend to be "overfitted", with many gene markers correlated to tumor categories purely by chance. To minimize this impact, rigorous validation of input mRNA quality is essential. This can be accomplished before microarray hybridization by electrophoretic methods[67,68] or by quantitative RT-PCR analyses that target 3' and 5' regions of control genes. Quality may also be estimated from Affymetrix data after hybridization, using a variety of parameters including 3':5' ratios; novel algorithms have been applied in studies of kidney and other cancers[69]. Furthermore, technical and biological replicates are incorporated into most studies to the extent feasible, in order to increase statistical confidence[70,71]. However, due to cost and sample constraints, few tumor classification studies provide enough replicates to eliminate data overfitting. Therefore, correlation of genes and tumor types can be strengthened by meta-analysis of multiple, independent microarray studies[72]. Despite the fact that different studies may use distinct microarray platforms and protocols, recent work has shown that current protocols achieve strong interlaboratory and interplatform concordance[73]. Finally, to validate microarray data for tumor classification fully, the differential expression of selected genes is often confirmed with independent methods such as quantitative RT-PCR or immunohistochemistry[74,75]. Validation takes on added importance for solid tumors, such as RCC, which are composed of heterogeneous cell populations; in many solid tumor studies, immunohistochemistry has been used both to confirm differential expression and to localize gene products to specific cell types[74,76,77,78,79].

## COMPUTATIONAL DATA ANALYSIS

For tumor classification, gene expression profiles must be compared across multiple tumor specimens. The computational tools to achieve this are categorized broadly as supervised and unsupervised algorithms[80]. Unsupervised clustering is effective for class discovery (e.g., exploratory identification of novel diagnostic or prognostic subtypes). Average-linkage hierarchical clustering[81] has been used in many cancer studies. This algorithm clusters tumors and genes into hierarchies defined by expression similarity, using metrics such as Pearson correlation. In many studies using hierarchical clustering, the tumor and gene clusters are displayed in dendrograms associated with color-coded grids that indicate relative overexpression or underexpression of each gene in each tumor specimen.

Supervised methods are ideal for class prediction (e.g., classifying unknown tumors into a previously established diagnostic subtypes) or identification of novel biomarkers. In supervised clustering, tumors are classified *a priori*, based on factors independent of the microarray data (e.g., diagnosis, therapy, clinical outcome), and genes are ranked by correlation with the supervised subclasses. The Significance Analysis of Microarrays (SAM) program is a common supervised algorithm, which uses random sample permutation to calculate a false discovery rate (ratio of false to total significant genes, averaged over all permutations) in order to estimate statistical significance[82]. Many studies have used supervised analyses to define “molecular classifiers”, consisting of smaller gene lists with strong correlation to tumor categories, which can predict identity of unknown tumor samples. Potential classifiers are tested for predictive power using computational methods such as “leave-one-out cross validation”. Leave-one-out algorithms remove individual tumors from the data set iteratively, and predict their identity by expression of genes in candidate classifiers. Molecular classifiers are optimized for rates of correct classification of left-out samples, and tested prospectively in independent sets of microarray data. Validated classifiers represent candidate targets for limited molecular assays applicable in clinical diagnostic laboratories.

### Knowledge-Based Analysis

Knowledge-based analysis frameworks[83] correlate microarray data with results of independent research, focusing attention on data likely to be valid and significant. Comparison of independent data is facilitated by large public data repositories with standardized annotation and formatting protocols, as well as detailed functional annotation of arrayed gene sequences using hierarchical controlled vocabularies[84,85,86,87,88]. Several statistical algorithms are now available to correlate differential expression profiles with the Gene Ontology controlled vocabulary[89,90]. Collectively, these knowledge-based tools can determine if tumor gene expression is significantly enriched or depleted for specific functional categories.

## DIAGNOSTIC CLASSIFICATION OF RENAL TUMORS

Using a variety of sample types and microarray platforms, several groups have shown that gene expression profiling is feasible for renal tumor classification, in a complementary manner to histopathology[74,75,76,77,78,79]. These analyses have provided consistent evidence that renal tumor subtypes, as defined by the WHO system, are characterized by unique gene expression profiles relevant to pathobiology and potentially significant for clinical diagnosis and management. In our recent study, gene expression was measured in clear cell RCC, papillary RCC, chromophobe RCC, oncocytoma, and angiomyolipoma using Affymetrix oligonucleotide microarrays. Unsupervised hierarchical algorithms clustered the tumors into groups that correlated strongly with histopathologic subtypes. Supervised data analysis with SAM, followed by functional classification based on Gene Ontology, revealed that clear cell RCC overexpressed immune response and angiogenesis genes, while chromophobe RCC and oncocytoma overexpressed energy pathway genes, including many genes associated with mitochondrial biology. Angiogenic gene expression may be related to tumor vascularity in clear cell RCC[2], while energy pathway gene expression may correlate with the abundant mitochondria in chromophobe RCC and oncocytoma[52]. Other groups have produced concordant findings[77].

Microarray data are consistent with current models of renal tumor histogenesis, which relate clear cell and papillary RCC to proximal nephron epithelium, and chromophobe RCC and oncocytoma to distal nephron intercalated cells[2]. In our experiments, clear cell RCC overexpressed the proximal nephron markers megalin and cubilin; papillary RCC strongly overexpressed the proximal nephron marker alpha methylacyl CoA racemase; and chromophobe RCC and oncocytoma overexpressed the distal nephron markers beta defensin-1, parvalbumin, chloride channel Kb, claudin-7, claudin-8, and epidermal growth factor. These findings have been validated in our laboratory by quantitative RT-PCR and immunohistochemistry, and are consistent with research from other laboratories. Thus, proximal and distal nephron markers may be useful for diagnostic classification of renal tumors.

The Van Andel Research Institute group has also published extensively on diagnostic classification of renal tumors by gene expression profiling. This group has utilized high-density spotted cDNA microarrays to identify subtype-specific expression profiles[78] and develop a comparative genomics microarray analysis (CGMA), which infers cytogenetic abnormalities from regional expression biases between tumors and adjacent non-neoplastic tissue. In a recent study, abnormalities inferred by CGMA correlated with recurring cytogenetic lesions in renal tumor subtypes. Furthermore, diagnostic classification of clear cell, papillary, and chromophobe RCC was strengthened by combining CGMA data with differential gene expression profiles[93]. Recent work from this group suggests that expression profiles and CGMA are sufficient to diagnose the subtypes of a large percentage of renal tumors prospectively, when correlated with previous microarray data[94]. The German Cancer Research Center group compared expression profiles with direct cytogenetic data from a large cohort of renal tumors, and confirmed that expression levels of specific genes correlated with the copy number of respective chromosomal arms.

To date, the principal impact of gene expression profiling on renal tumor diagnosis has been through discovery of novel immunohistochemical markers for each major tumor subtype. Table 1 summarizes renal tumor immunomarkers identified with microarrays. We have shown that a panel of beta defensin-1, parvalbumin, and vimentin is superior to any single marker used alone for classification of clear cell RCC, papillary RCC, and chromophobe RCC/oncocytoma[79]. The distinction between chromophobe RCC and oncocytoma remains challenging, but is clinically important since the former has malignant potential while the latter is benign. In all microarray studies thus far, expression profiles of these subtypes are remarkably similar[74,78,91]. Correspondingly, it has been difficult to find immunohistochemical markers for this differential diagnosis. The RON oncogene and kidney-specific cadherin have been proposed in individual studies, but not confirmed in others[95,96,97,98]. In our recent microarray analysis, claudin-7 was identified as a rare sequence overexpressed in chromophobe RCC vs. oncocytoma, and immunohistochemistry supported this pattern[74]. Conversely, S-100 protein may be expressed preferentially in oncocytoma[99]. Additional studies are needed to validate these expression markers.

Tissue microarrays (TMAs) have emerged as an alternative approach to explore the utility of existing immunohistochemical antigens for renal tumor diagnosis. TMAs allow candidate markers to be evaluated in many tumor specimens simultaneously; parallel TMA sections can be used to study multiple markers with high efficiency. Several antigens have been proposed for renal tumor classification based on TMA analysis, including S-100[99] and cyclin D1, D3, E, and p27[100].

Quantitative, "real-time" RT-PCR is now available in many clinical diagnostic laboratories and is more quantitative than immunohistochemistry. We have developed assays for megalin, alpha methylacyl CoA racemase, beta defensin-1, chloride channel Kb, and other markers that utilize fixed tissue RNA and are able to distinguish the major subtypes of renal tumor reliably. An independent group has used this method to show that mRNA levels of parvalbumin, chloride channel Kb, alpha methylacyl CoA racemase, and carbonic anhydrase IX are useful for diagnostic classification[101]. The German Cancer Research Center group developed a promising 80-gene "molecular classifier" for renal tumors and validated several genes by quantitative RT-PCR. Larger prospective studies are needed to establish the clinical potential of immunohistochemistry and RT-PCR for renal tumor diagnosis.

**TABLE 1**
**Immunohistochemical Markers for Renal Tumor Classification that have been Discovered by Gene Expression Profiling**

| Marker | Gene Symbol | Target Tumor | Biological Relevance | Localization | Reference |
|---|---|---|---|---|---|
| Vimentin | VIM | Clear cell RCC | Cytoskeleton | Cytoplasm | Young et al.[79,91], Moch et al.[113] |
| Adipophilin | ADFP | | Cell differentiation | Cytoplasm | Yao et al.[75] |
| CD10 antigen | CD10 | | Neutral endopeptidase | Membrane | Higgins et al.[77], Avery et al.[114] |
| Glutathione S-transferase α | GSTA | | Cell detoxification | Cytoplasm | Takahashi et al.[78], Chuang et al.[76] |
| CD74 antigen | CD74 | | Immune response | Membrane | Young et al.[91], Saito et al.[115] |
| α Methylacyl CoA racemase | AMACR | Papillary RCC | Peroxisomal enzyme | Cytoplasm | Takahashi et al.[78], Tretiakova et al.[92] |
| Cytokeratin 7 | CK7 | | Cytoskeleton | Cytoplasm | Higgins et al.[77] |
| β Defensin-1 | DEFB1 | Chromophobe RCC and oncocytoma | Antimicrobial and antitumor activity | Cytoplasm | Young et al.[79,91] |
| Parvalbumin | PVALB | | Calcium-binding protein | Cytoplasm | Young et al.[79,91], Martignoni et al.[116] |
| Stem cell factor receptor | KIT | | Cell differentiation | Membrane | Higgins et al.[77], Petit et al.[117], Yamazaki et al.[112] |
| Carbonic anhydrase II | CA2 | | Zinc metalloenzyme | Cytoplasm | Takahashi et al.[78] |
| Claudin-7 | CLDN7 | | Cell adhesion | Membrane | Schuetz et al.[74] |

## PROGNOSTIC CLASSIFICATION OF RENAL TUMORS

The University of Texas group compared gene expression in localized RCC, metastatic RCC, and patient-matched non-neoplastic kidney[102]. Transforming growth factor beta type III receptor (TBR3) was underexpressed in all RCC cases, and type II receptor (TBR2) was underexpressed in metastatic RCC. These findings were confirmed in cell lines representative of metastatic and nonmetastatic RCC. Restoration of TBR3 and TBR2 expression in metastatic cell lines blocked cell proliferation and anchorage-independent growth. Thus, development and progression of RCC may involve stepwise lesions in transforming growth factor beta signaling.

The Van Andel Research Institute group analyzed 29 clear cell RCC and matched normal kidney associated with long-term clinical follow-up[103]. Patients were not matched for known prognostic indicators (e.g., grade, stage, or performance status); however, they were separated into two groups of distinct outcome by unsupervised clustering. Supervised analysis revealed gene subsets that correlated with cause-specific 5-year survival, independent of histologic grade and pathologic stage. Leave-one-out algorithms were used to validate a 40-gene classifier to predict outcome. In this classifier, transforming growth factor beta and downstream effectors were underexpressed in the poor outcome group, consistent with the University of Texas study[102], while angiogenesis inhibitors were overexpressed in the good outcome cohort. The National Cancer Institute group examined 58 cases of stage IV kidney cancer associated with good clinical performance status, but not stratified by histologic subtype[104]. Supervised data analysis and leave-one-out cross-validation revealed a 45-gene signature correlating with poor outcome, which did not overlap significantly with prognostic markers from the Van Andel Research Institute study. Vascular cell adhesion molecule 1 was identified as a candidate marker for good prognosis. The German Cancer Research Center group identified genes correlating with metastasis and survival, several of which have been proposed as prognostic markers previously. Collectively, these microarray studies indicate that gene expression may be useful to predict outcome in RCC, supporting established clinical and pathologic prognostic factors, pending validation in prospective studies on independent RCC cohorts.

Several biomarkers have been studied as prognostic tools for RCC using immunohistochemistry. For example, adipophilin, which was discovered initially as a marker of clear cell RCC by micorarray analysis[75,91], was shown to be associated with favorable outcome in this tumor subtype by immunohistochemistry[75]. Building on previous TMA analysis, it was recently shown that relative cyclin B1 expression and aberrant cytoplasmic localization in tumor vs. adjacent benign tissue correlated with higher-grade, nodal metastasis and poor prognosis[105].

The Wyeth Research group compared gene expression in peripheral blood mononuclear cells (PBMCs) of healthy volunteers vs. advanced RCC patients enrolled in a clinical trial of the Wyeth drug CCI-779 (temsirolimus; a mammalian target of rapamycin, or mTOR, inhibitor). Unsupervised clustering distinguished the PBMCs of most RCC patients from those of healthy volunteers. Since circulating tumor cells could confound these results, the investigators determined that informative genes from the PBMC samples did not correspond to those associated previously with renal carcinoma cells. A training subset of the study cohort was analyzed with supervised algorithms to develop an expression classifier for distinguishing RCC patients from healthy volunteers. This classifier was validated with leave-one-out algorithms on the training subset, followed by predictive confirmation of the remaining cohort (testing subset)[106]. A similar approach was used to identify gene expression markers for predicting patient outcome after CCI-779 therapy. The performance of gene expression was comparable to standard methods for clinical risk classification[19]. This research highlights a potential for minimally invasive assays on peripheral blood to complement RCC diagnosis, therapeutic management, and clinical trial design.

## THERAPEUTIC IMPLICATIONS OF GENE EXPRESSION PROFILING

Many overexpressed genes in RCC tumor tissue have therapeutic implications. Clear cell RCC overexpresses immune response genes, as shown with several systems including expression microarrays[74,91]. This expression profile may be important for the relative responsiveness of clear cell RCC to immunotherapy[48,107]. Expression levels of immune response genes vary among individual tumors[91] and future studies should determine if variability correlates with response to immunotherapy. As a promising example of this scenario, a group from Friedrich-Schiller University used expression microarrays to discover overexpression of the lymphocyte activator antigen CD70 in clear cell RCC vs. benign kidney; differential expression was confirmed by quantitative RT-PCR and immunohistochemistry[108]. An multi-institutional group subsequently showed that CD70 expression mediated sensitivity of RCC cells to anti-CD70 antibody-drug conjugates *in vitro* and in nude mouse xenografts[109].

Angiogenesis genes are overexpressed in clear cell RCC, likely related to VHL and HIF1A dysregulation. This distinct expression profile is expected to be significant for responsiveness to the various antiangiogenic therapies being evaluated in clinical trials[49]. Angiogenic and immune response regulators both increase expression of carbonic anhydrase IX, which is the target of G250 monoclonal antibody therapy for RCC. In large microarray studies of multiple cancers, it appears that RCC may be distinguished from other tumor types by overexpression of angiogenesis genes and coregulation of VEGF and carbonic anhydrase IX[110].

The dysregulation of HIF1A results in activation of multiple angiogenic tyrosine kinase molecules, such as VEGF and platelet derived growth factor (PDGF)[42], and also appears to influence response to the mTOR inhibitor CCI-779/temsirolimus[40]. Based on these observations, large clinical trials have assessed the activity of temsirolimus, as well as the multikinase inhibitors sunitinib and sorafenib in metastatic RCC[41,42,111]. These novel molecular-based therapies appear to inhibit disease progression with acceptable toxicity profiles, in comparison to standard cytokine therapy. Microarray studies have established that stem cell factor receptor (KIT) is overexpressed in chromophobe RCC[77,112], leading several experts to suggest tyrosine kinase inhibitors such as Gleevec for advanced carcinomas of this subtype.

## CONCLUSIONS

Molecular classification by gene expression profiling has begun to make significant contributions for diagnosis, prognosis, and treatment of RCC. In the diagnostic setting, the growing use of small tumor biopsies in minimally invasive surgery has emphasized the need for novel molecular diagnostic markers that complement histopathology. For clinical management, the continued study of well-documented RCC patient cohorts with metastatic phenotypes, and long-term survival and therapeutic outcome data, is likely to promote development of novel assays for patient management. In particular, markers to predict recurrence after surgery or systemic therapy, or to assess risk of metastasis to specific sites, would be valuable tools to tailor management according to prognosis. As methods for functional analysis of microarray data continue to improve, microarray studies are likely to contribute fundamentally to knowledge of renal tumor biology in order to guide selection of rational targets for molecular therapy and disease monitoring.

## ACKNOWLEDGMENTS

Supported by NCI Grant 1 U54 CA119338-02 (ANY).
